# Beyond reducing direct medical cost: examining health outcomes in tuberculosis through a difference-in-differences analysis of South Korea’s out-of-pocket payment exception policy

**DOI:** 10.3389/fpubh.2024.1380807

**Published:** 2024-05-23

**Authors:** Sarah Yu, Daseul Moon, Dawoon Jeong, Young Ae Kang, Gyeong In Lee, Hongjo Choi

**Affiliations:** ^1^School of Health Policy and Management, College of Health Science, Korea University, Seoul, Republic of Korea; ^2^BK21 FOUR R&E Center for Learning Health Systems, Korea University, Seoul, Republic of Korea; ^3^Busan Center for Infectious Disease Control and Prevention, Busan, Republic of Korea; ^4^Department of Preventive Medicine, Seoul National University College of Medicine, Seoul, Republic of Korea; ^5^Division of Pulmonary and Critical Care Medicine, Department of Internal Medicine, Severance Hospital, Yonsei University College of Medicine, Seoul, Republic of Korea; ^6^The Korean Institute of Tuberculosis, Korean National Tuberculosis Association, Cheongju, Republic of Korea; ^7^Department of Preventive Medicine, Konyang University College of Medicine, Daejeon, Republic of Korea

**Keywords:** health outcomes, out-of-pocket payment, universal health coverage, tuberculosis, treatment completion

## Abstract

**Background:**

Universal health coverage and social protection are major global goals for tuberculosis. This study aimed to investigate the effects of an expanded policy to guarantee out-of-pocket costs on the treatment outcomes of patients with tuberculosis.

**Methods:**

By linking the national tuberculosis report and health insurance data and performing covariate-adjusted propensity-score matching, we constructed data on health insurance beneficiaries (treatment group) who benefited from the out-of-pocket payment exemption policy and medical aid beneficiaries as the control group. Using difference-in-differences analysis, we analyzed tuberculosis treatment completion rates and mortality in the treatment and control groups.

**Results:**

A total of 41,219 persons (10,305 and 30,914 medical aid and health insurance beneficiaries, respectively) were included in the final analysis (men 59.6%, women 40.4%). Following the implementation of out-of-pocket payment exemption policy, treatment completion rates increased in both the treatment and control groups; however, there was no significant difference between the groups (coefficient, −0.01; standard error, 0.01). After the policy change, the difference in mortality between the groups increased, with mortality decreasing by approximately 3% more in the treatment group compared with in the control group (coefficient: −0.03, standard error, 0.01).

**Conclusion:**

There are limitations to improving treatment outcomes for tuberculosis with an out-of-pocket payment exemption policy alone. To improve treatment outcomes for tuberculosis and protect patients from financial distress due to the loss of income during treatment, it is essential to proactively implement complementary social protection policies.

## 1 Introduction

Universal health coverage (UHC) has been discussed as a core strategy for resolving public health issues related to tuberculosis (TB) (1). UHC is a state in which everyone can receive necessary medical services for health improvement, prevention, treatment, rehabilitation, and alleviation without suffering excessive financial hardships, and is one of the major goals and strategies of public healthcare (2). UHC reduces diagnostic delay, increases treatment acceptance, and improves treatment outcomes by improving accessibility to high-quality healthcare and minimizing out-of-pocket (OOP) payments. Thus, UHC can improve public healthcare in various ways, including reducing the incidence, prevalence, disability rate, and mortality rates (3, 4).

UHC comprises three dimensions: population, services, and financial protection. By implementing the national health insurance for the entire population in 1989, Korea has achieved the population dimension. However, the total healthcare coverage ratio, derived from the covered services and percentage of costs covered, remains low. Health service coverage is only 63.4% owing to the high OOP payment ratio and an increase in uninsured services; the 36.8% OOP health expenditure rate in Korea is 20.5% higher than the Organization for Economic Co-operation and Development (OECD) average. Moreover, the proportion of catastrophic health expenditures increased from 1.6% in 2000 to 4.5% in 2015 [OECD average in 2016, 0.7% (5)]. Therefore, improving health insurance coverage remains an important task in national public healthcare planning. Policies are being established to improve coverage, focusing on major diseases, treatment costs, social strata, and non-reimbursable services (6). One such policy is the “OOP exception policy,” which aims to reduce medical costs for diseases requiring expensive or lengthy treatment. With the expansion of OOP exception policy, TB, which also requires a long-term treatment, had OOP payments reduced from 30 to 10% in 2009 and was completely eliminated in 2016. In other words, by exempting patients from all costs for TB-related medical services, the OOP exception policy has partially achieved UHC.

Strategies to achieve a UHC differ depending on the characteristics of each country, including their socioeconomic environment; the indices used to evaluate the effects of these strategies are also diverse (7). United States lacks a public health insurance system; therefore, the debate has been dominated with discussions on Medicaid expansion (the Affordable Care Act), focusing on increasing population coverage. Thus, the effects of the system are reported in measures such as expanded coverage, improved accessibility to medical services, and improved health conditions (8–11). In middle-to-low-income countries, OOP for healthcare systems are actively used to reduce catastrophic health expenditure, and the effects are assessed in terms of OOP reduction, lower incidence, and increase in positive health conditions (12, 13) In Korea, where a nationwide health insurance system is used, many studies have evaluated the effects of OOP exception policy in increasing medical cost coverage; however, the most commonly used outcome indices have been copayments for medical expenses and the rate of experiencing insufficient healthcare, whereas its effects on health improvement has not been widely studied (6, 14, 15).

Furthermore, although UHC, which can be achieved by minimizing the likelihood of financial hardship, can effectively improve the health of the population, its effects have not been sufficiently considered from a comprehensive standpoint. This study aimed to investigate the effects of UHC on health improvement by analyzing the effects of OOP exception policy on treatment outcomes in Korean patients with TB.

## 2 Methods

### 2.1 Study design and population

This retrospective cohort study investigated TB treatment outcomes in health insurance and medical aid beneficiaries after the implementation of the OOP payments exception policy for patients receiving treatment for TB. We used data from the Korean National Tuberculosis Surveillance System and National Health Information Database from 2013 to 2018. We combined the two datasets using resident registration numbers and selected all patients diagnosed with one of the following International Classification of Diseases 10^th^ revision codes: A15–A19, B90, or U84.3. Data were anonymized before constructing the retrospective cohort. For other specific methods, we followed the methodology described in a previous study (16). This study was conducted in accordance with the 2008 Declaration of Helsinki and was approved by the appropriate Institutional Review Board of Severance Hospital (4–2019-0917). The need for informed consent was waived due to the retrospective nature of the study.

**Figure 1:**
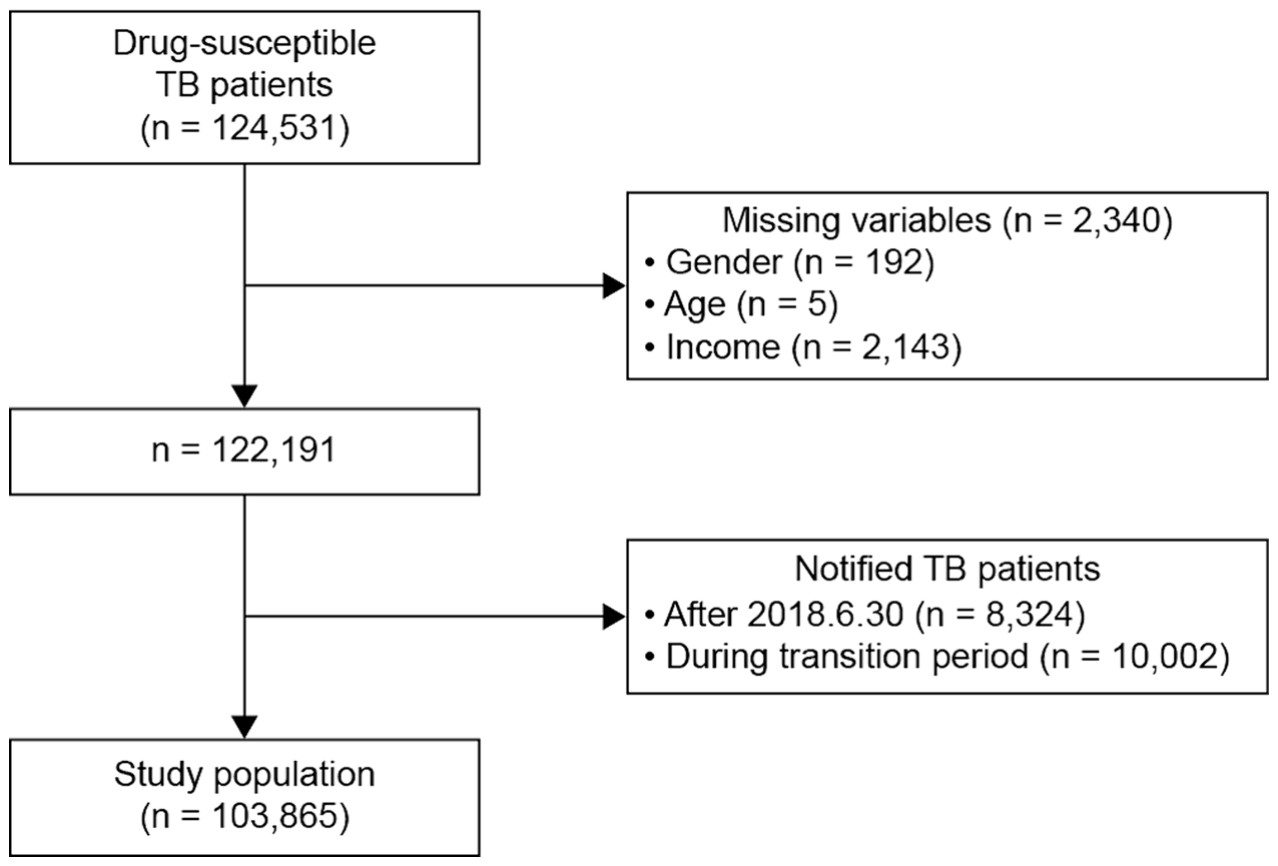
Flow diagram of the study population. TB, tuberculosis.

Patients with TB between 2013 and 2018 were included in this study. The exclusion criteria were as follows: patients (1) with drug-resistant TB; (2) whose treatment outcomes could not be easily verified (patients reported after June 30, 2018); and (3) who were reported during the transition time between policies (July 1 –December 31, 2016). Of the 124,531 patients with drug-sensitive TB reported between 2013 and 2018, 103,865 were included in the final analysis (Figure 1). A total of 2,340 patients were excluded because of missing data on gender, age, or income (192, 5, and 2,143 patients, respectively); 8,324 and 10,002 patients were excluded owing to difficulties in verifying treatment outcomes and because they were reported during the transition period, respectively. In the final cohort, we included 6,545 medical aid and 60,417 health insurance beneficiaries from before the policy introduction and 3,759 medical aid and 33,143 health insurance beneficiaries from after the policy introduction (Figure 1).

### 2.2 Measures

National health insurance beneficiaries were defined as those who benefited from the policy. Although Korea has a health insurance system for the entire population, National health insurance subscriptions are limited to population in the top 97% of incomes. For those in poverty (i.e., the bottom 3% of incomes), a separate medical aid program is provided, allowing them to receive medical services without having to pay an insurance fee (17). Because the OOP exception policy applies to health insurance beneficiaries, we set them as the treatment group and medical aid beneficiaries as the control group.

The policy of interest was the OOP exception policy, which was implemented on July 1, 2016 and exempted patients with TB from previous OOP payments for 10% of the medical costs. The period before policy implementation was defined as July 1, 2013 to December 31, 2015, and the period after policy implementation was defined as July 1, 2016 to December 31, 2018.

The outcome variables of interest were the treatment completion rate and mortality, which are the main TB treatment outcomes. The treatment outcomes were classified as death, completion, and non-completion. Death was defined as patients who died owing to TB-related or other causes within 2 years of starting TB treatment, and completion was defined as consuming at least 80% of the TB medication during the treatment period. Cases that did not meet the death or completion definitions were considered non-completion (suspension of treatment or awaiting evaluation). The treatment completion rate was calculated as the number of patients who completed treatment relative to the number of patients reported with TB, excluding those who died during treatment. Mortality was calculated as the number of patients who died of all causes within 2 years of starting TB treatment relative to the total number of patients reported with TB.

Basic demographics including age, gender, disability, income, comorbidities, and characteristics related to TB were examined as covariates. Comorbidities included malignant tumors, terminal kidney disease, and diabetes. TB-related characteristics included smear test results (negative/positive), TB classification (pulmonary/extrapulmonary), and TB history. Patients with missing data on age, gender, or income were excluded from the analysis.

### 2.3 Statistical analysis

To compare the characteristics of the two groups before and after policy implementation, we performed chi-square tests for each control variable. We performed propensity-score matching (PSM) to ensure that the distribution of baseline characteristics was similar between the two groups. We used age, gender, and year of TB onset for PSM to achieve a 1:3 matching of medical aid beneficiaries to health insurance beneficiaries. Subsequently, we performed a propensity score-matched difference-in-differences analysis using two-way fixed-effects (TWFE) regression (18).


$$\begin{array}{c} Y=\alpha +\beta_{1}\mathrm{Treatment}+\beta_{2}\mathrm{Post}+\beta_{3}\left(\mathrm{Treatment}\times \mathrm{Post}\right)\\ +\overset{n}{\underset{k=1}{\sum }}\beta_{k}\mathrm{Covariate}+\varepsilon \end{array}$$

Y represents a binary variable (treatment completion/non-completion or death/survival); the post-time point is defined as 0 for pre-policy (January 2013–December 2015) or 1 for post-policy (July 2016–December 2018), and treatment is defined as whether the patient benefited from the policy (medical aid beneficiary = 0, health insurance beneficiary = 1). The covariates included gender, age, disability status, income level, smear test results, TB classification, TB history, and major comorbidities (malignant tumors, terminal kidney disease, and diabetes). All the reported *p*-values were two sided, and p-values <0.05 were considered statistically significant. Data manipulation and statistical analyses were conducted using Stata version 17 (StataCorp, College Station, TX, United States).

## 3 Results

### 3.1 Patient characteristics

A total of 103,865 patients with TB were included in the analysis (10,305 in the control and 93,560 in the treatment groups). When we inspected the demographic characteristics of each group before PSM, the control group was older than the treatment group and showed a higher rate of mild and severe disability (*p*-value<0.01; Table 1). After PSM, 30,914 health insurance beneficiaries were included in the analysis, and no significant differences were observed between the groups in terms of age, year of TB report, or smear test results. However, the likelihood of disability and comorbidities remained slightly higher in the control group.

**Table 1:** Characteristics of study population before and after propensity-score matching.

Variable	Medical aid beneficiary (*n* = 10,305)		Health insurance beneficiary					
			Before PSM (*n* = 93,560)			After PSM (*n* = 30,914)		
	*n*	%	*n*	%	*p*-value	*n*	%	*p*-value
Age group					<0.001			>0.99
<14	26	0.3	504	0.5		97	0.3	
15–19	194	1.9	2,640	2.8		580	1.9	
20–24	172	1.7	3,941	4.2		498	1.6	
25–29	95	0.9	4,438	4.7		285	0.9	
30–34	118	1.2	4,690	5.0		354	1.2	
35–39	169	1.6	4,996	5.3		507	1.6	
40–44	319	3.1	6,325	6.8		957	3.1	
45–49	658	6.4	7,178	7.7		1,974	6.4	
50–54	963	9.3	8,420	9.0		2,889	9.4	
55–59	1,124	10.9	9,283	9.9		3,372	10.9	
60–64	866	8.4	7,503	8.0		2,598	8.4	
65–69	761	7.4	6,448	6.9		2,283	7.4	
70–74	961	9.3	7,594	8.1		2,883	9.3	
≥75	3,879	37.6	19,600	21.0		11,637	37.6	
Gender					0.81			0.83
Men	6,135	59.5	55,812	59.7		18,443	59.7	
Women	4,170	40.5	37,748	40.4		12,471	40.3	
Disability					<0.01			<0.01
None	6,650	64.5	83,180	88.9		26,505	85.7	
Mild	1,426	13.8	6,238	6.7		2,723	8.8	
Severe	2,229	21.6	4,142	4.4		1,686	5.5	
Notification year					0.01			>0.99
2013	2,474	24.0	21,735	23.2		7,446	24.1	
2014	2,151	20.9	20,053	21.4		6,467	20.9	
2015	1,921	18.6	18,629	19.9		5,763	18.6	
2016	1,003	9.7	8,959	9.6		3,009	9.7	
2017	1,864	18.1	16,647	17.8		5,568	18.0	
2018	892	8.7	7,537	8.1		2,661	8.6	
Type of notification institution					0.34			0.80
Public	131	1.3	1,297	1.4		383	1.2	
Private	10,174	98.7	92,263	98.6		30,531	98.8	
Smear test					<0.01			0.07
Negative	6,812	66.1	72,876	70.0		20,738	67.1	
Positive	3,493	33.9	31,196	30.0		10,176	32.9	
History of TB					<0.01			<0.01
New case	7,742	75.1	78,073	83.5		24,862	80.4	
Relapse	2,563	24.9	15,487	16.6		6,052	19.6	
TB classification					<0.01			<0.01
Pulmonary	8,668	84.1	73,974	79.1		30,231	97.8	
Extrapulmonary	1,637	15.9	19,586	20.9		683	2.2	
Comorbidities								
Malignancy					0.70			0.18
No	10,100	98.0	91,751	98.1		30,231	97.8	
Yes	205	2.0	1,809	1.9		683	2.2	
Kidney failure					<0.01			<0.01
No	10,074	97.8	92,639	99.0		30,566	98.9	
Yes	231	2.2	921	1.0		348	1.1	
Transplantation					0.02			0.04
No	10,282	99.8	93,438	99.9		30,874	99.9	
Yes	23	0.2	122	0.1		40	0.1	
Diabetes mellitus					<0.01			<0.01
No	6,490	63.0	73,566	78.6		22,287	72.1	
Yes	3,815	37.0	19,994	21.4		8,627	27.9	
Treatment result					<0.01			<0.01
Not completion	1,759	17.1	10,852	11.6		3,698	12.0	
Completion	6,766	65.7	75,353	80.5		23,637	76.5	
Death	1,780	17.3	7,355	7.9		3,579	11.6	

PSM, propensity-score matching; TB, tuberculosis.

Treatment outcomes were significantly more negative in the control group than in the treatment group, both before and after PSM. Before PSM, the treatment completion rate was 80.5% in the treatment group, which was higher than in the control group (65.7%), whereas the negative treatment outcomes of non-completion and mortality among the treatment group (11.6 and 7.9%) were lower than those in the control group (17.1 and 17.3%), respectively. After PSM, the control group showed a higher rate of negative treatment outcomes than the treatment group, but the difference was smaller. Mortality in the treatment group increased from 7.9 to 11.6% and the completion rate decreased from 80.5 to 76.5%.

### 3.2 Difference-in-differences

**Figure 2:**
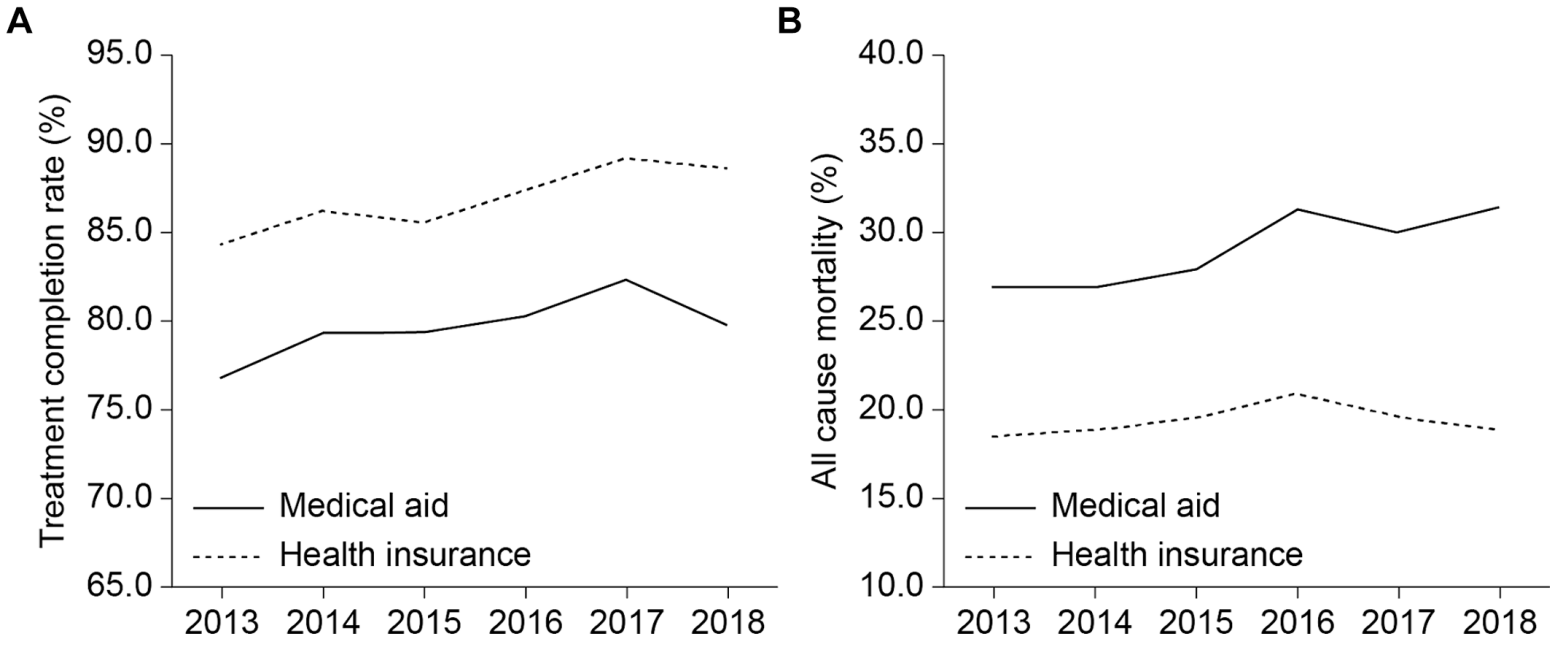
Trends in outcomes among the treatment and control groups: **(A)** treatment completion rate (%) and **(B)** all-cause mortality.

We examined annual changes in the treatment completion and mortality rates of patients reported within the study period (Figure 2). The treatment group showed a higher treatment completion rate and lower mortality rate than the control group. Before the policy implementation, the control (solid line) and treatment groups (dotted line) showed similar trends. However, after OOP exception policy introduction, especially between 2017 and 2018, the treatment completion rate of the control group declined rapidly compared with that of the treatment group. Moreover, although mortality rates decreased in the treatment group, the rates increased in the control group.

After the introduction of OOP exception policy for patients with TB, the effects of reduced OOP payments for TB treatment, with the treatment completion rate as the outcome variable, were not observed in the treatment group in the difference-in-differences analysis (Table 2). The difference in completion rates between the treatment and control groups was statistically significant before and after the policy was introduced, irrespective of correction for covariates (all *p* < 0.01). However, the difference-in-differences effect for the interaction between time and group was not statistically significant both before or after correcting for covariates (before corrections, *p* = 0.62; after corrections, *p* = 0.26).

**Table 2:** TB treatment success rate in medical aid and health insurance beneficiary TB patients before and after the OOP exception policy.

	Crude analysis			Adjusted model^a^		
	Coefficient	SE	*p*-value	Coefficient	SE	*p*-value
Before policy						
Control group	0.78			0.52		
Treatment group	0.85			0.67		
Difference (A)	0.07	0.01	<0.01	0.15	0.01	<0.01
After policy						
Control group	0.81			0.61		
Treatment group	0.89			0.74		
Difference (B)	0.07	0.01	<0.01	0.14	0.01	<0.01
B − A	0.01	0.01	0.62	−0.01	0.01	0.26

^a^ Covariates: gender, age, disability, household income, type of notification institution, result of smear, type of tuberculosis, history of TB, comorbidities (malignancy, kidney failure, diabetes mellitus).

Control group: medical aid beneficiaries; treatment group: health insurance beneficiaries.

OOP, out-of-pocket; SE, standard error; TB, tuberculosis.

Conversely, in the difference-in-differences analysis with mortality as the outcome variable, the difference between the treatment and control groups increased significantly after policy introduction (*p* < 0.01; Table 3). The coefficient for mortality in each cohort showed a positive value that gradually increased before correcting for covariates but showed a negative value that gradually decreased after corrections.

**Table 3:** Mortality rate of medical aid and health insurance beneficiary TB patients before and after the OOP exception policy.

	Crude analysis			Adjusted model^a^		
	Coefficient	SE	*p*-value	Coefficient	SE	*p*-value
Before policy						
Control group	0.27			−0.29		
Treatment group	0.19			−0.35		
Difference (A)	−0.08	0.01	<0.01	−0.06	0.01	<0.01
After policy						
Control group	0.31			−0.27		
Treatment group	0.20			−0.36		
Difference (B)	−0.11	0.01	<0.01	−0.09	0.01	<0.01
B − A	−0.03	0.01	<0.01	−0.03	0.01	<0.01

^a^ Covariates: gender, age, disability, household income, type of notification institution, result of smear, type of tuberculosis, history of TB, comorbidities (malignancy, kidney failure, diabetes mellitus).

Control group: medical aid beneficiaries; treatment group: health insurance beneficiaries.

OOP, out-of-pocket; SE, standard error; TB, tuberculosis.

## 4 Discussion

### 4.1 Effect on treatment outcomes

This study aimed to investigate the effects of OOP exception policy on the treatment outcomes of patients with TB. Our findings demonstrated the limited effects of the OOP exception policy, which aims to support direct medical costs for patients with TB. Irrespective of the policy, inequality in treatment completion rate and mortality remained between the treatment and control groups. The OOP exception policy introduction did not improve treatment completion rate in the treatment group. However, this policy decreased the mortality rate in the treatment group by approximately 1%.

### 4.2 Financial implications of medical costs

Following the OOP exception policy implementation, the treatment completion rate in the treatment group did not increase. This result contradicted previous discussions in which medical costs were highlighted as the main cause of long-term treatment interruption among patients with TB and the need to support medical costs was proposed (19). Lee et al. (15) conducted an interrupted time-series analysis on differences in TB treatment outcomes (i.e., the long-term treatment interruption rate and mortality) following the OOP exception policy introduction among Korean health insurance beneficiaries and reported findings similar to ours. Specifically, after the policy implementation, patients with drug-susceptible TB showed a decrease in long-term treatment interruption only in the continuation phase but not in the intensive phase. In patients with drug-resistant TB, interruptions increased in the intensive phase after the policy introduction, and no change was observed in the continuation phase. To illustrate a UHC system, the OOP exemption policy alleviates the financial hardship due to direct medical expenses. However, health insurance beneficiaries could still experience financial hardship due to nonmedical costs (4).

### 4.3 Non-medical costs and income loss

Furthermore, medical costs are not the only problem associated with TB treatment. As emphasized in strategies to end TB, long-term treatment is required for patients with TB, and social costs are a major factor affecting failure to continue treatment (20). These findings are consistent with previous results showing positive treatment outcomes (treatment success, treatment completion, and microbiologic cure) when patients were provided with social protection, such as cash transfer, travel reimbursement, transportation subsidy, and food security, in addition to medical costs (21, 22). Another study analyzed the outcomes of a free TB treatment policy in Burkina Faso using different indices of TB treatment failure (i.e., sputum-smear microscopy, chest X-ray, hospitalization, additional tests) and concluded that achieving the final aims of UHC (i.e., improving TB treatment) by removing user fees alone is difficult (23). To our knowledge, the largest proportion of financial consequences of TB were related to the non-medical cost such as food and nutritional supplement costs and income loss according to the global surveys (24). Furthermore, housing would be a challenge for vulnerable population such as homeless population. However, those might not be eliminated with the OOP exemption policy only. South Korea is conducting a pilot study to launch the paid sickness leave policy from 2022, which could reduce income loss due to TB. Moreover, housing and food provision was identified an effective intervention in the Korean context (25). Therefore, it would be a time to consider more policy beyond the OOP exemption policy for TB patients.

### 4.4 Disparities and equity

Although the OOP exception policy did not affect treatment completion rate, it partially contributed to the decrease in mortality rate in the treatment group. This finding was consistent with previous results, such as those of a comparative study that demonstrated a negative correlation between the UHC Service Coverage Index and TB mortality (26), and a Korean study using survival analysis to investigate mortality after the OOP exception policy introduction (15). An even more notable aspect of our results was that following the policy introduction, mortality rate inequality between the two groups increased because mortality rate was not only decreased in the treatment group but also increased in the control group. These results were consistent with another important finding from our study: both treatment outcomes were more successful in the treatment group than in the control group, irrespective of the policy introduction. The control group consisted of Koreans in the lowest 3% income bracket who use medical services through a tax-relief-based medical aid system that is separate from the Korean National health insurance system. This medical aid system is divided into type 1 beneficiaries, who are guaranteed completely free access to all insured services, and type 2 beneficiaries, who must make copayments. Both groups still have to pay medical costs for uninsured services. This shows that although this group had most of their medical costs covered before and after the policy implementation, they could still experience a financial burden owing to poverty and low economic status. Patients with TB who received medical aid showed a lower rate of visiting medical institutions and lower prescription rates (TB quality assessment) than patients with TB covered by health insurance. These findings indicated that Korean UHC does not properly account for the value of “equity.” We cannot exclude the possibility that, although the service was available to everyone universally, the control group might have fallen even further behind in terms of service provision. Consequently, the mortality rate increased in this group.

### 4.5 Study strengths

The strength of this study was that we constructed a TB patient cohort by integrating data from the Korean National Tuberculosis Surveillance System and National Health Information Database, allowing us to include all patients with TB in Korea. Accordingly, we were able to resolve the statistical error discussed in a recent difference-in-differences study using TWFE regression analysis. The difference-in-differences estimates from the TWFE regression require the assumption that the treatment effects are the same over time and also consistent among subpopulations in the treatment group. However, in reality, outcomes change over time after the policy introduction (27). When panel data are obtained through sampling and analysis, observations are made for a long period after treatment to increase statistical power; thus, difference-in-differences estimates using TWFE regression are not appropriate (28). In the present study, we avoided these problems by constructing a cohort of patients with TB based on the National Health Information Database.

### 4.6 Study limitations and future recommendations

Our study has some limitations in respect of the outcome measurements. We used the treatment completion rate as a surrogate for treatment success because it was not feasible to directly calculate the cure rates using the available claims’ data. Consequently, the treatment success rate may be either overestimated or underestimated. Furthermore, our study considered all-cause deaths as one of the outcome. In addition, there were fundamental differences between medical aid beneficiaries and health insurance beneficiaries, such as income level. Our analysis was unable to reduce this difference due to choosing a control population unaffected by the policy. Alternatively, we applied PSM to minimized other confounders. In future, research to enhance precision, it may be advisable to include TB-related deaths as a specific outcome.

## 5 Conclusion

To achieve effective UHC, it is essential to introduce social protection policies for loss of income alongside support for direct medical costs and consider equity when implementing UHC. As has been heavily discussed previously, to achieve the end of TB, it is necessary to consider UHC and universal social protection including the paid sickness leaves and housing supports, which aims to provide financial protection from a nonmedical perspective. However, direct and indirect costs are not the only problems associated with TB treatment and its outcomes. Given the effects of gender, employment status, poverty, health behavior, attitudes, and associated social inequality, developing and implementing TB policies from the perspective of social determination remain necessary.

## Data availability statement

Publicly available datasets were analyzed in this study. This data cannot be shared publicly because of the regulations of the National Health Insurance Service. Data are available from the Review Board of the National Health Insurance Service (contact via NHIS) for researchers who meet the criteria for access to confidential data. Applications for data are available through National Health Insurance Data Sharing website (https://nhiss.nhis.or.kr/bd/ab/bdaba000eng.do), and additional information can be found at a customized health information data webpage (https://nhiss.nhis.or.kr/bd/ab/bdaba032eng.do).

## Ethics statement

The studies involving humans were approved by the Institutional Review Board of Severance Hospital (4–2019-0917). The studies were conducted in accordance with the local legislation and institutional requirements. The ethics committee/institutional review board waived the requirement of written informed consent for participation from the participants or the participants’ legal guardians/next of kin because the need for informed consent was waived due to the retrospective nature of the study.

## Author contributions

SY: Writing – original draft. DM: Writing – original draft. DJ: Formal analysis, Investigation, Methodology, Software, Validation, Visualization, Writing – original draft. YK: Funding acquisition, Project administration, Resources, Supervision, Writing – review & editing. GL: Funding acquisition, Project administration, Resources, Writing – review & editing. HC: Conceptualization, Formal analysis, Investigation, Methodology, Validation, Visualization, Writing – original draft, Writing – review & editing.
